# A *Botrytis cinerea KLP-7* Kinesin acts as a Virulence Determinant during Plant Infection

**DOI:** 10.1038/s41598-017-09409-5

**Published:** 2017-09-06

**Authors:** Pamil Tayal, Sumit Raj, Esha Sharma, Manoj Kumar, Vikram Dayaman, Nidhi Verma, Abhimanyu Jogawat, Meenakshi Dua, Rupam Kapoor, Atul Kumar Johri

**Affiliations:** 1 Department of Botany, University of Delhi, Delhi, 110 007 India; 2 School of Life Sciences, Jawaharlal Nehru University, Delhi, 110 067 India; 3 School of Environmental Sciences, Jawaharlal Nehru University, New Delhi, 110 067 India

## Abstract

*Botrytis cinerea* is a necrotrophic pathogen that infects many important crops. In an attempt to unravel some novel factors that govern pathogenicity in *B. cinerea*, *Agrobacterium tumefaciens* mediated transformation (ATMT) was deployed, and a number of tagged transformants were generated. Among these, a mutant, BCM-29 exhibited slower growth rate, reduced conidia size, conidiation and penetration. The mutant was also defective in secretion of oxalic acid (OA) and exhibited reduced activities of polygalacturonase (PG) and pectin methyl esterases (PME). TAIL-PCR followed by BLAST search identified the tagged gene as *KLP-7* that encodes for kinesin. Targeted deletion of *KLP-7* resulted in several folds decrease in virulence of mutants as compared to WT, while complementation of the gene helped in rescue of virulence traits. This is the first time when a unique kinesin *KLP-7* that is mainly found in the phylum Pezizomycotina has been linked to virulence in *B. cinerea*.

## Introduction

*Botrytis cinerea* Persoon ex. Fries (teleomorph: *Botryotinia fuckeliana* (de Bary) Whetzel) is a widely distributed phytopathogen, that causes serious pre and post harvest yield losses in more than 235 different plant species in a range of agronomically important crops such as strawberry (*Fragaria ananassa*), tomato (*Solanum lycopersicum)*, grapevine (*Vitis vinifera* ***)***, cucumber (*Cucumis sativus*). It affects almost all vegetative parts such as bulb, stem, leaves and even flowers of ornamental plants under conducive conditions^1, 2^. The fungus is widely distributed in the temperate areas of the world where it infects an extremely wide range of host plants^3^. The stages of plant infection by *B. cinerea* includes attachment of conidia on host surface, germination i.e. germ tube formation, penetration and colonization that finally leads to killing of the host tissue resulting in tissue maceration^4, 5^.

*B. cinerea* is capable of attacking crops at all stages of their growth and under storage; also affects all plant parts^6^. *B. cinerea* germ tubes enter the plant *via* direct penetration through natural openings or wounds to derive nutrients from dead or decaying cells^7^; therefore its colonization in the plant depends upon the ability of the fungus to kill the host cells. Chemical control is the primary means to reduce the incidence of this pathogen. However, the technique is only partially successful as it develops resistance against a wide range of fungicides. Therefore, it is vital to understand the biology of pathogen, and study host-pathogen interaction for the development of improved strategies for effective disease resistance.

A large number of candidate genes for virulence like tetraspanin - *BcPls*1 factor, Mitogen-Activated Protein (MAP) kinase - *BMP*1 and components of some signalling transduction cascade have been identified in *B. cinerea* using targeted gene inactivation approach^8–17^. In addition, several virulence factors like cell wall degrading enzymes, transporter proteins and enzymes for protecting the fungus from oxidative stress are also involved in symptom development^18–23^. In parallel, random insertional mutagenesis approach has also yielded a wealth of pathogenicity mutants that led to identification of new, partly unexpected virulence factors^24, 25^.

In an attempt to unravel some novel factors that govern pathogenicity in *B. cinerea*, we utilized ATMT and generated a number of tagged transformants. A mutant namely BCM-29 that showed considerably reduced pathogenicity was selected for detailed characterization. Tagged gene in the mutant was identified via TAIL-PCR and BLAST analysis. The sequence showed homology to a gene *KLP-7* that encodes a kinesin protein.

Kinesins are microtubule based energy hydrolysing motor proteins that help in the segregation of genetic material during cell division and cell growth. A high number of kinesin sub-families exist in the angiosperms, however, only a few kinesins have been identified in fungi. Schoch *et al*.^26^, identified a total of 11 kinesin in *B. fuckeliana*. Based on parsimony analysis of the alignment, it was suggested that “*KLP-7*p clade constitutes a unique fungal subgroup of “truncated” UNC-104-like proteins that may constitute a new subfamily”. Though in fungi, kinesins have been reported to play an important role in morphogenesis of fungal hyphae, however, their consequent effect on virulence of a pathogen has not been studied so far. To corroborate the role of fungal *KLP-7* in virulence, we have applied ATMT, gene tagging, complementation and targeted gene deletion strategies. We found that *KLP-7* is involved in the regulation of hyphal growth, conidia germination, tissue penetration that suggests its role in pathogenicity of *B. cinerea*.

## Results

### ATMT

**Figure 1:**
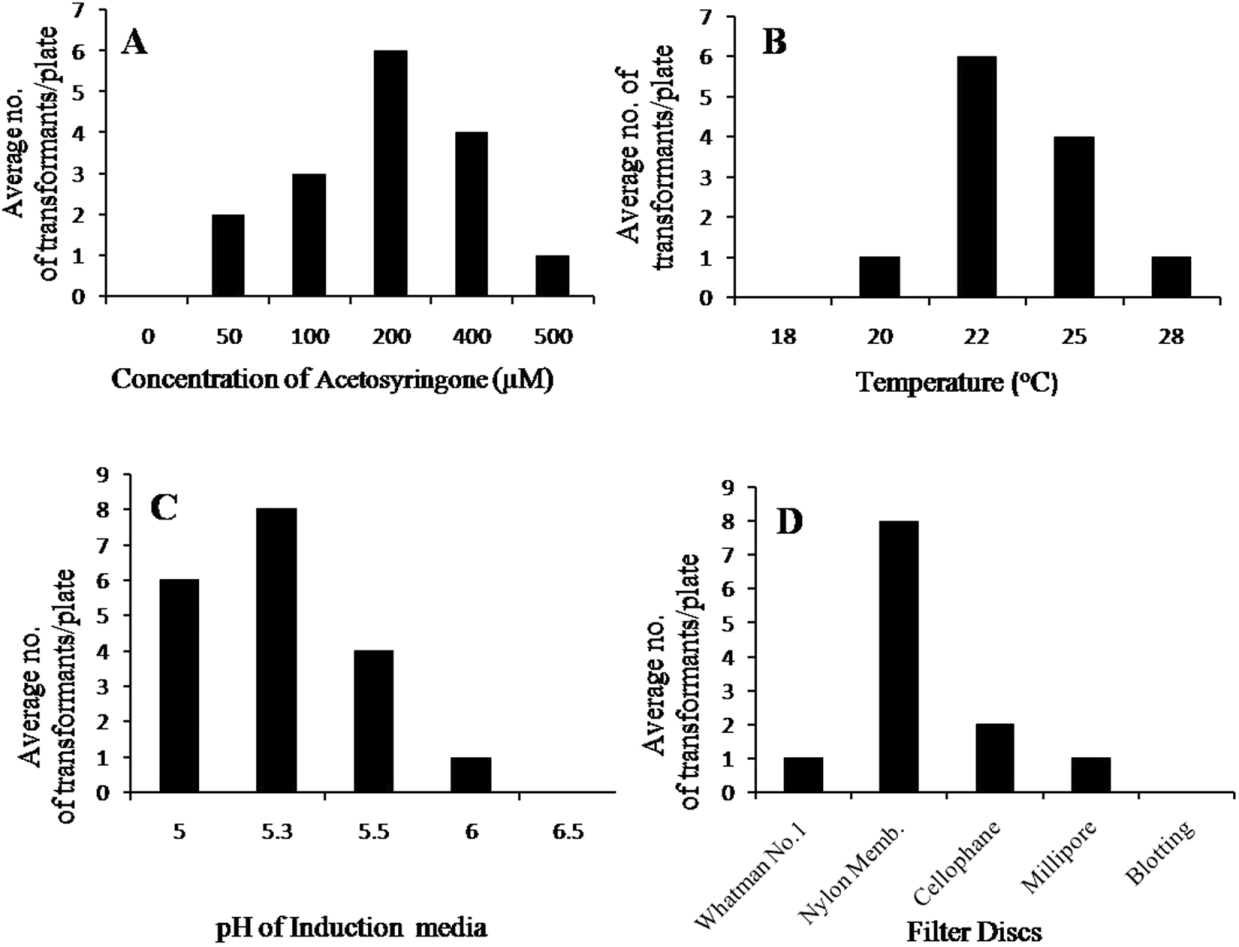
Optimization of co-cultivation conditions for *Agrobacterium tumefaciens* mediated transformation of *B. cinerea*. (**A**) Concentration of acetosyringone (µM); (**B)** Temperature of co-cultivation; (**C**) pH of Induction medium; (**D)** Filter discs.

Successful random insertional mutagenesis of WT was achieved by using LBA 4404 strain of *A. tumefaciens*. The optimised protocol generated 8–12 transformants per 1 × 10^5^ conidia (Fig. 1A–D). The transformation efficiency was good when 1:1 ratio of *A. tumefaciens* and *B. cinerea* spores was used. The maximum number of transformants were obtained when 200 µM of As was used, although transformation efficiency decreased with further increase in concentration of As (Fig. 1A). Maximum transformation efficiency was achieved at 22 °C. It was observed that at 25 °C and 28 °C, the transformation efficiency reduced to four and one transformants (on an average) respectively (Fig. 1B). The effect of pH during co-cultivation on the transformation efficiency was also tested. No transformants were obtained at pH 6.5. Decrease in pH to 6.0 or 5.5 resulted in one and four transformants respectively, while a further decrease in pH to 5.3 resulted in maximum number of 8 transformants (Fig. 1C). Different filters such as Whatman No. 1, Millipore filter discs, Nylon membrane and blotting filter paper were used to test the efficiency of T-DNA transfer. Maximum transformants were obtained on Nylon membrane (pore size, 0.22 µm). However, Millipore filter discs with the same pore size were not found efficient for *B. cinerea* transformation (Fig. 1D).

A total of 800 transformants were raised using this approach. Initial pathogenicity screening led to the identification of 200 transformants with a range of defects. However, after multiple rounds of pathogenicity screening, one transformant, BCM-29 showed consistent impaired pathogenicity on chickpea plants. Per cent disease severity of WT and BCM-29 was 86.6% and 17.9% respectively. Considering the significant impairment (79.4%) in pathogenicity, the transformant was selected for further characterization.

### Characterization of T-DNA tagged mutant BCM-29

**Figure 2:**
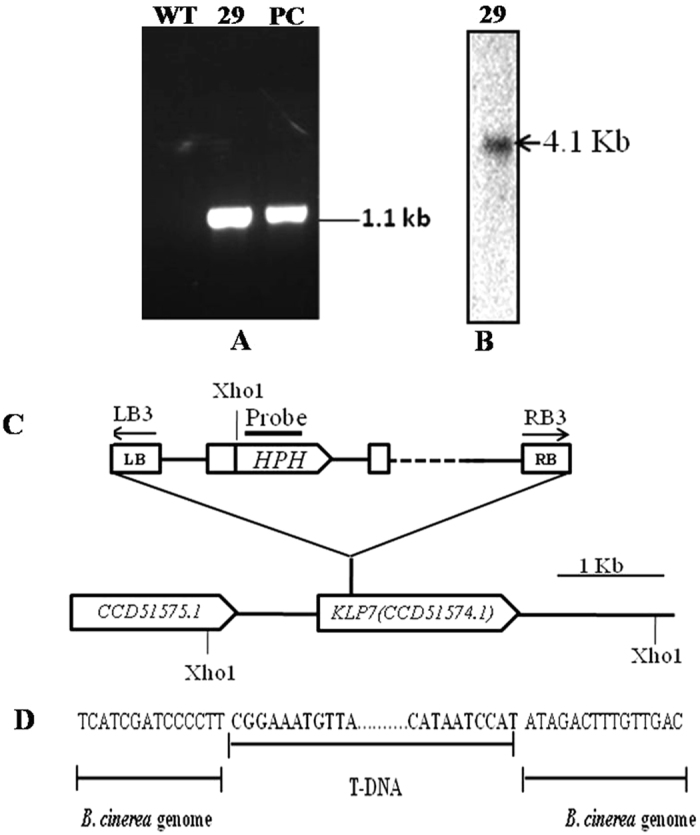
An abnormal T-DNA integration in *KLP-7*. **(A)** Hygromycin phosphotransferase (*hph*) gene insertion in BCM-29. The expected PCR product size is 1.1 kb. Lane 1, Ladder of 10 kb; lane 2, WT, lane 3, BCM-29, lane 4, positive control (PC) (*hph*) (**B)**. Southern hybridization showing the integration of 4.1 kb fragment of *hph* gene in genome of BCM 29. Total genomic DNA was digested with *Xho*I and probed with the *hph* probe; (**C)** Schematic presentation integration of T-DNA in BCM 29. Specific primers were used for the confirmation of T-DNA insertion shown by arrows. The T-DNA insertion point is 116 bp from *KLP-7* start codon (**D)** Insertion of T-DNA in *B. cinerea* genome. Junction site are shown in bold letters.

Integration of *hph* gene in BCM-29 was confirmed by PCR analysis using *hph* specific primers. An amplicon (1.1 kb) corresponding to the selectable marker gene cassette was detected in the mutant (Fig. 2A). Further genomic DNA of BCM-29 was subjected to Southern hybridization to determine the T-DNA copy number. A single band of 4.1 kb was observed when genomic DNA of BCM-29 hybridized with the *hph* probe which confirmed that insertional mutant was produced due to single copy T-DNA integration in the genome of *B. cinerea* (Fig. 2B). The mutant BCM-29 retained its mitotic stability as it could grow proficiently on medium supplemented with hygromycin even after five successive generations of growth in the absence of antibiotic selection (data not shown).

T-DNA tagged gene in the mutant was identified by TAIL-PCR. Amplification of flanking sequence of BCM-29 was performed on both left and right borders using border specific primers (RB or LB) along with an arbitrary degenerate primer (AD). In the primary PCR cycle multiple bands were visible that disappeared after successive secondary and tertiary PCR reactions, indicating that the disappeared bands were non-specific. In tertiary PCR a single band was observed (Fig. S1) and the recovered sequences corresponded to right border junction. The sequences on BLAST analysis were found to be similar to the supercontig 10_4 of the *B. cinerea* genome. Schematic diagram of the T-DNA integration in BCM 29 is shown in (Fig. 2C). Junction sequences between the T-DNA and the *B. cinerea* genome revealed that the T-DNA had a typical RB border at one end. The T-DNA was located 116 bp downstream from the start codon of *KLP-7* ORF (Fig. 2D).

### KLP-7 encodes kinesin gene

**Figure 3:**
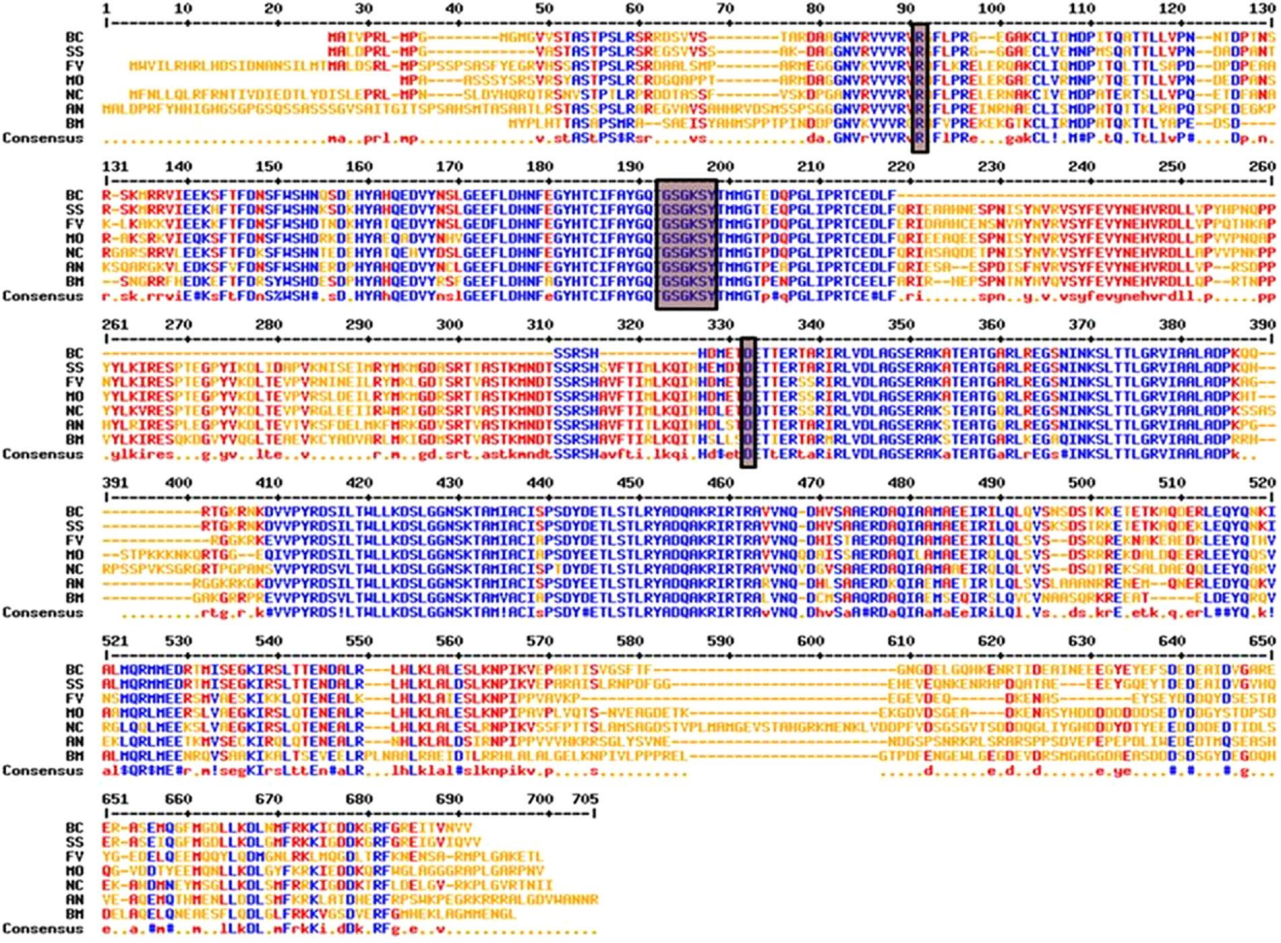
Alignment of the deduced amino acid sequence of *KLP-7. B. cinerea* AAO59283 (BC) with *Sclerotinia sclerotiorum* XP_001598213 (SS); *Fusarium verticillioides* EWG48122 (FV), *Magnaporthe oryzae* XP_003715125 (MO), *Neurospora crassa* XP_961491(NC), *Aspergillus niger* XP_001401241(AN), *Bipolaris maydis* AAO59294 (BM) by using MULTALIN. The high degree of sequence conservation at each position of amino acids is shown in blue, and low consensus amino acids are shown in red. Signature tag sequences of conserved motor domain of KIF1A/UNC4 are shown in box.

**Figure 4:**
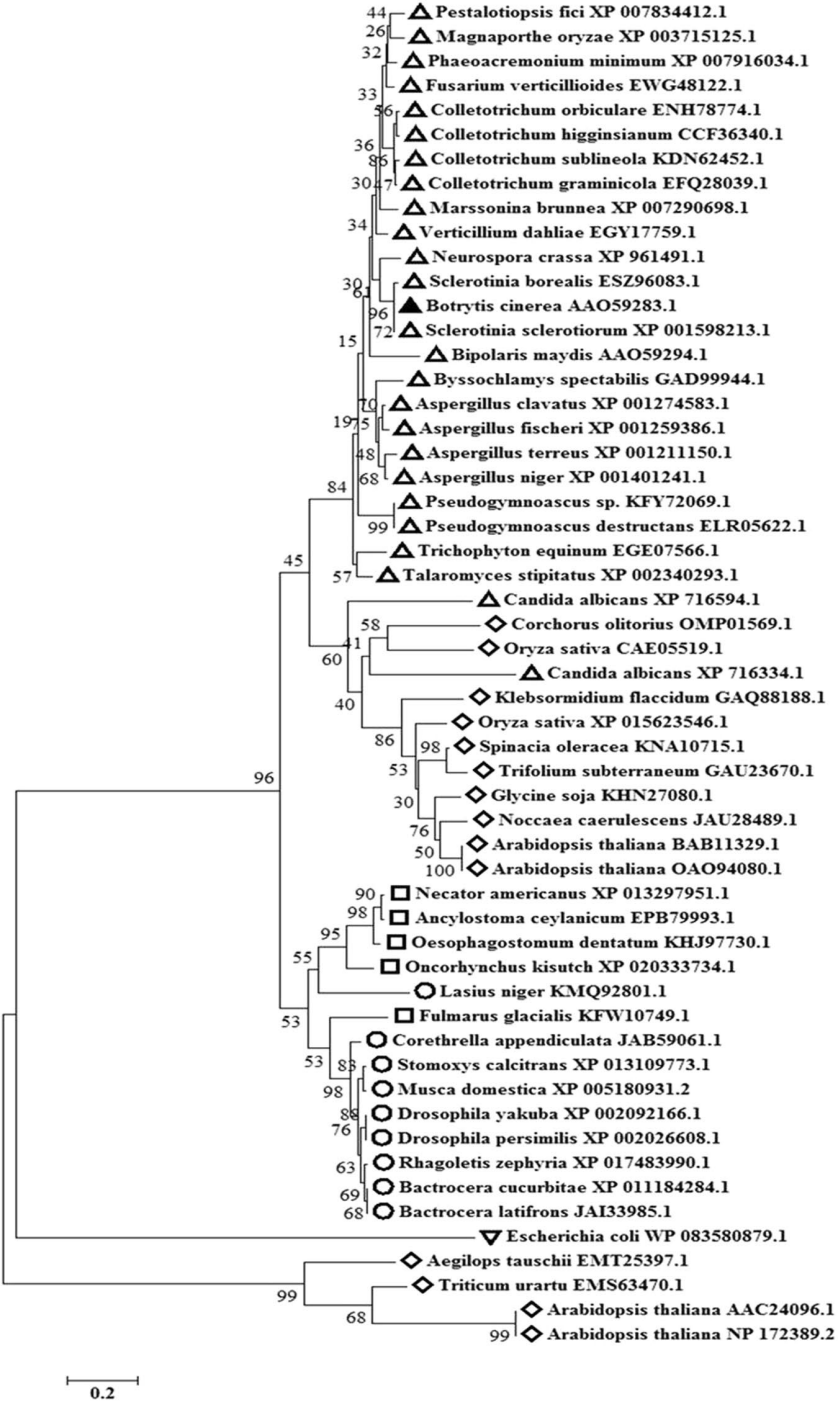
Phylogenetic analysis: The evolutionary history was inferred using the Neighbor-Joining method. The optimal tree with the sum of branch length = 8.63069435 is shown. Percentage of replicate trees in which the associated taxa clustered together in the bootstrap test (1000 replicates) are shown next to the branches. Tree is drawn to scale, with branch lengths in the same units as those of the evolutionary distances used to infer the phylogenetic tree. Evolutionary distances were computed using the Poisson correction method and are in the units of the number of amino acid substitutions per site. Analysis involved 55 amino acid sequences. All positions containing gaps and missing data were eliminated. There were a total of 78 positions in the final dataset. Evolutionary analyses were conducted in MEGA7. Member of different groups were marked with different shape i.e. Δ: fungi, □: animals, ◊: Plants, ○: Insects, ∇: Bacteria and *B. cinerea* kinesin protein is marked with filled triangle (▲) shape to display its position.

Putative *KLP-7* gene was found to be 1,491 bp long, encoding 496 amino acids (GenBank submission ID: 1779638) **(**Fig. S2 **)**. BLAST search showed that putative *KLP-7* has homology with the motor domain of kinesin of filamentous fungi (Table S1). Interestingly, *KLP-7* homologs were found only in subphylum Pezizomycotina of Ascomycota (Table S1). In all the fungal species belonging to Pezizomycotina, the *KLP-7* homologs were highly conserved (Table S1). They share more than 60% similarity in their amino acid sequences. Our MULTALIN analysis showed that putative *KLP-*7 has a conserved signature tag (R)-(G)-(GKSY)-(D) of cd01365 of kinesin motor domain, KIF1_like proteins. The motor domain was found at the N-terminal with catalytic (head) domain. In contrast to KIF1A/Unc104 kinesins, lysine-rich loop were not observed in *KLP-7* that binds to the negatively charged C-terminus of tubulin and compensates for the lack of a second motor domain (Fig. 3). A circular phylogenetic tree demonstrates the conserved *KLP-7* proteins in fungal members of the pezizomycotina having conserved motor domain with similar length (Fig. S3). An amino acid level similarity of *B. cinerea* KLP-7 kinesin with other diverse organisms’ kinesin-like proteins is shown in (Table S2 **)**. Maximum similarities of KLP-7 kinesin were observed with phytopathogenic fungi specifically from Ascomycota as compared to other organisms such as plants, insects, bacteria and other animals (Table S2). A neighbor joining phylogenetic tree analysis revealed that KLP-7 Kinesin stands among fungal members of subphylum Pezizomycotina (Phylum: Ascomycota) (Fig. 4).

### Complementation of T-DNA tagged BCM-29

**Figure 5:**
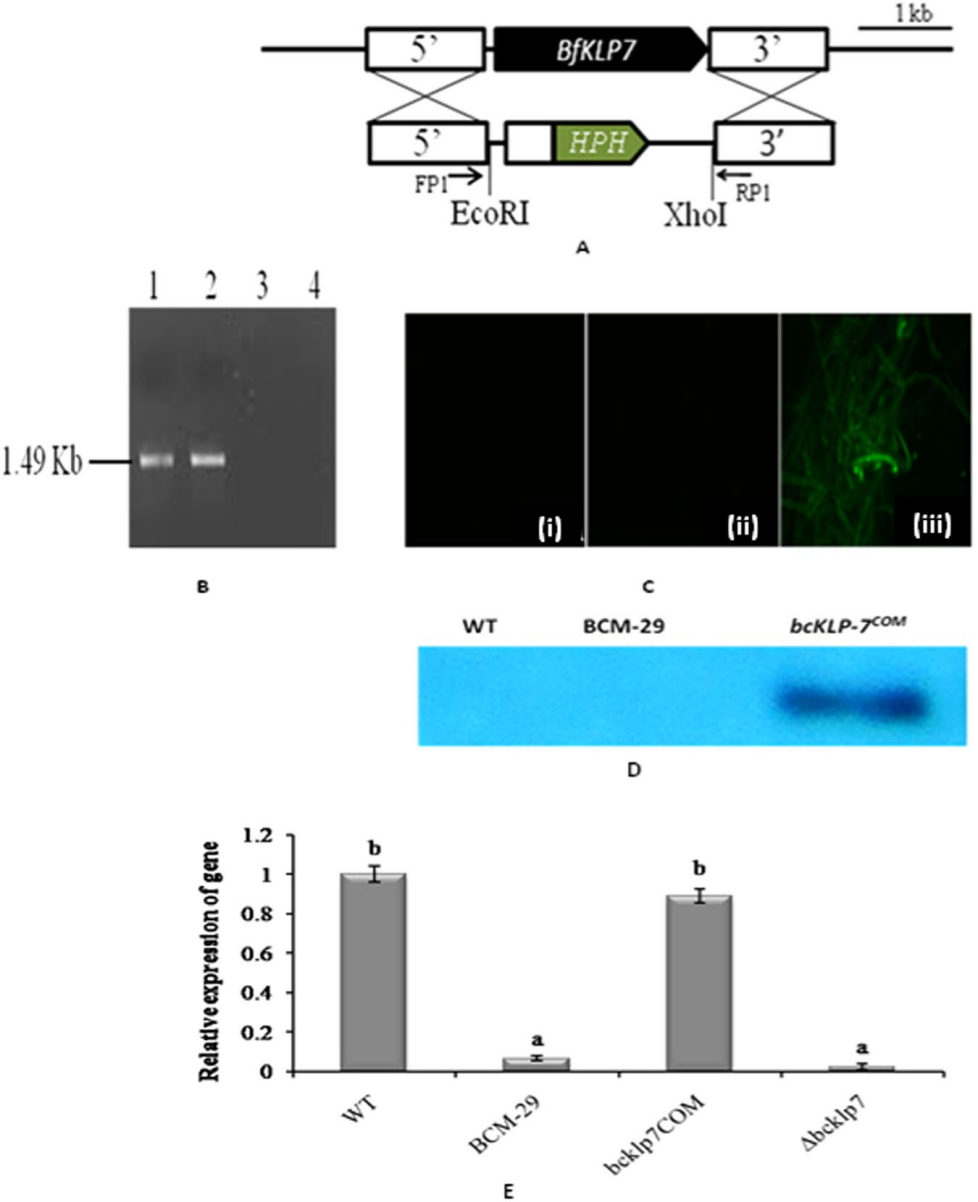
Identification of a *KLP-7* deletion mutant in *B. cinerea* and relative gene expression analysis. (**A**) The *KLP-7* deletion cassette (3,571 bp) with the *hph* cassette replaced the *bcKLP-7* ORF by double crossing over. Flanking genomic regions are shown by white box (**B**) Gel electrophoretic profile showing amplification of *KLP-7* gene using gene specific primer pair in WT and mutants. Lane1: WT, Lane 2: complemented mutant *bcKLP-7* ^COM^, Lane 3: T-DNA tagged mutant BCM-29, Lane 4: deletion mutant *∆bcKLP-7* (**C**) Fluorescence microscopy for the expression analysis of GFP in (i) WT, (ii) BCM-29 and (iii) *bcKLP-7* ^COM^ (**D**) Western blot analysis for GFP expression in WT, BCM-29 and *bcKLP-7* ^COM^ (**E**) Expression analysis of *KLP-7* in WT, BCM-29, *bcKLP-7* ^COM^ and in *∆bcKLP-7*. Relative expression levels were normalized to the mean of the expression of reference gene (actin). The expression of WT was set to 1. Data is presented as mean ± SD (n = 3). Bars showing different letters indicate significant differences between WT and mutants according to the Duncan’s multiple comparison tests (P < 0.05).

PCR analysis showed a presence of 1.49 kb fragment in case of *bcKLP-7* ^COM^ which confirmed the ectopic integration of *KLP-7* in the genome (Fig. 5B lane 2). In case of BCM-29, no band of *KLP-7* gene was observed (Fig. 5B lane 3).

### Targeted deletion of putative KLP-7

Gene deletion *via* ATMT was carried out in WT to ascertain its role in fungal pathogenesis. Amplification of 1.49 kb gene in WT was observed by PCR (Fig. 5B lane 1). No gene amplification was observed in case of ∆*bcKLP-7* which confirmed the successful deletion of *KLP-7* gene (Fig. 5B lane 4). The mutant was also confirmed on the basis of *hph* gene amplification (data not shown).

### GFP expression analysis

The insertion of putative *KLP-7* gene in *bcKLP-7* ^COM^ was also confirmed by fluorescence microscopy. No GFP expression was observed in case of WT and BCM-29, however a strong presence of GFP was observed in case of *bcKLP-7* ^COM^ (Fig. 5C, i-iii). Western blot analysis showed a strong band of GFP in case of *bcKLP-7* ^COM^, however no band was observed in case of WT *B. cinerea* and BCM-29, which confirmed the transformation (Fig. 5D).

### Expression analysis of KLP-7

Gene expression of putative *KLP-7* was examined in WT, BCM-29, *bcKLP-7* ^COM^ and ∆*bcKLP-7* by qRT-PCR. Significantly higher expression level of putative *KLP-7* was observed in case of WT. We have observed ten and eleven fold higher gene expression in WT and *bcKLP-7* ^COM^ respectively, as compared to BCM-29. In case of ∆*bcKLP-7*, *KLP-7* expression reduced significantly as compared to WT and *bcKLP-7* ^COM^ and was found to be similar to BCM-29 (Fig. 5E).

### Characterization of mutants

**Figure 6:**
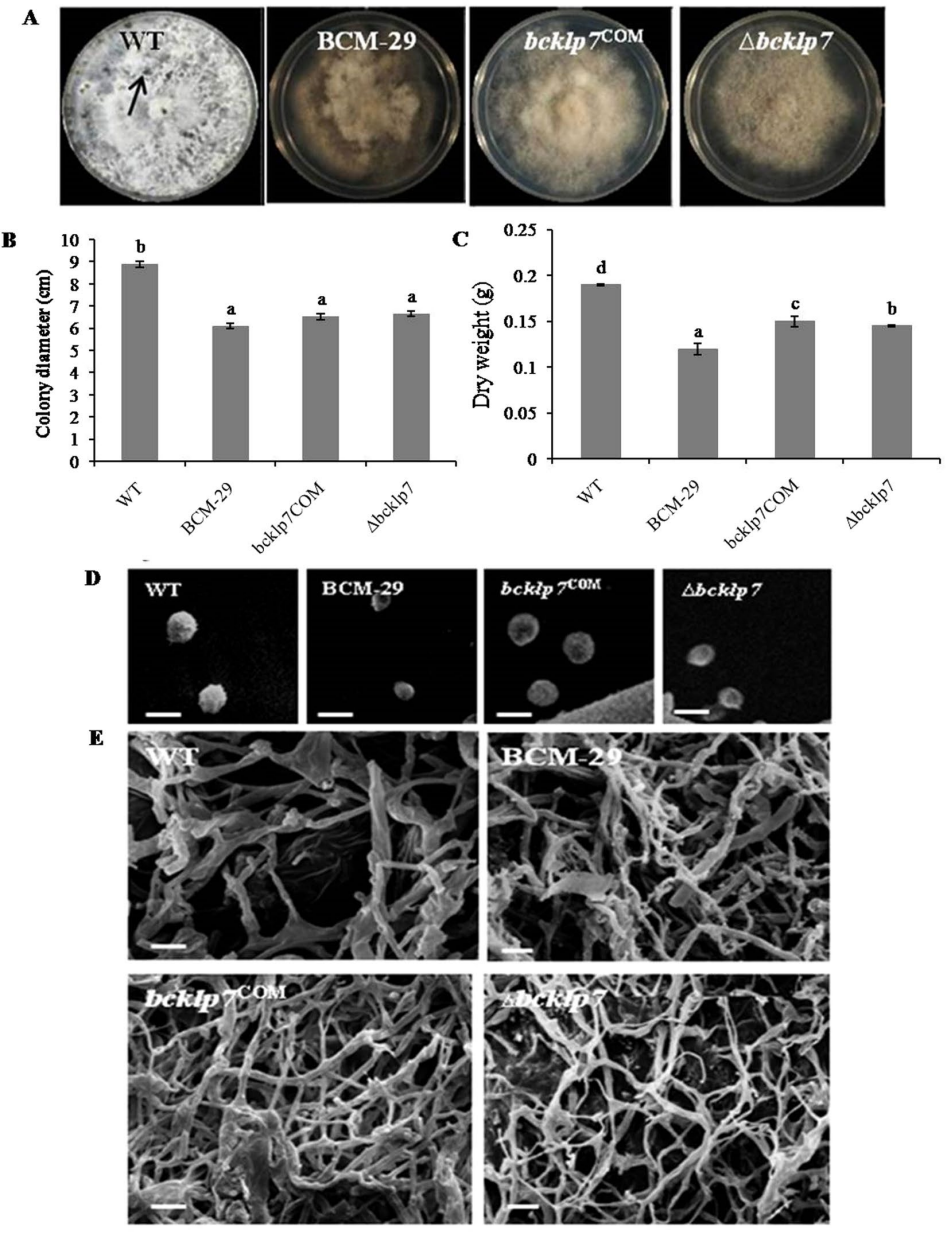
Radial growth, sclerotia formation, mycelia dry weight assay and scanning electron microscopy of conidia and hyphal structure. (**A**) Colony morphology and development of sclerotia. The wild type strain, BCM-29, *bcKLP-7* ^COM^ and *∆bcKLP-7* were incubated on PDA medium at 22 °C for 3 weeks in darkness. Arrow indicates the melanised structure, sclerotia; (**B**) Colony diameters of WT, BCM-29, *bcKLP-7* ^COM^ and *∆bcKLP-7* after 7 days growth on PDA medium; (**C**) The dry weight mycelium assay for WT and mutants. Cultures were incubated in Potato dextrose broth for seven days. Data is presented as mean ± SD. Bars showing different letters indicate significant differences between WT and mutants according to the Duncan’s multiple comparison tests (P < 0.05); (**D**) Conidia morphology and size observed under scanning electron microscope; (**E**) Hyphal structure of indicated strains observed under a scanning electron microscope. Scale bar represents 10 µm. Data is presented as mean ± SD. Bars showing different letters indicate significant differences between WT and mutants according to the Duncan’s multiple comparison tests (P < 0.05).

Phenotypically, WT, BCM-29, *bcKLP-7* ^COM^ and *∆bcKLP-7* appeared cottony white and turned grey black with age. The sclerotia (resting, melanised structures produced under dark) formation was observed in WT only (Fig. 6A). In contrast to WT, BCM-29, *bcKLP-7* ^COM^ and ∆*bcKLP-7* showed significant slower growth rate (P < 0.05). While the mean colony diameter decreased significantly (P < 0.05) in case of all the three mutants as compared to WT, no significant difference was observed among them (Fig. 6B). Impact of reduced growth was also observed on dry weight of the mycelium of mutant strains. Variations were observed among all the strains, with highest dry weight recorded in WT followed by *bcKLP-7* ^COM^ (Fig. 6C).

### KLP-7 affects conidia size, hyphae growth and sporulation

The scanning electron micrographs showed that WT and mutants produced numerous small hyaline conidia. However, the size of conidia varied among all the strains. Conidia were bigger in WT and *bcKLP-7* ^COM^ as compared to BCM-29 and ∆*bcKLP-7* (Fig. 6D). Hyphal morphology was also affected. Hyphae were uniform (smooth) and broad in WT as compared to BCM-29 and ∆*bcKLP-7* which showed irregular and constricted hyphae with more branches as compared to WT and *bcKLP-7* ^COM^ (Fig. 6E). Conidia production was also lesser in BCM-29 and ∆*bcKLP-7* as compared to that of WT and *bcKLP-7* ^COM^ (data not shown).

### Pathogenicity assay for *B. cinerea* mutants (BCM-29, *bcKLP-7*^*COM*^ and *∆bcKLP-7*)

**Figure 7:**
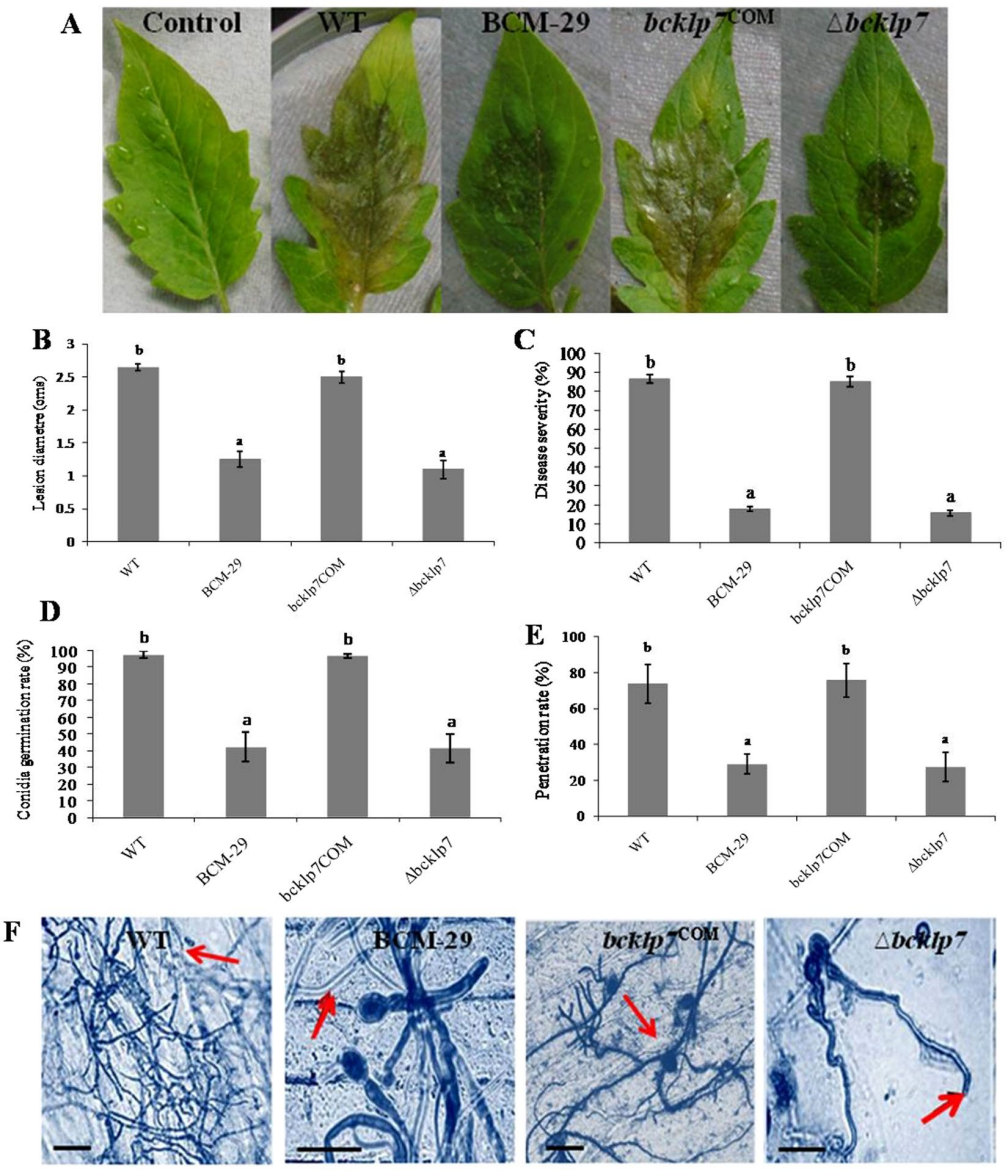
Infection related morphogenesis and role of virulence components. **(A)** Virulence assay on detached tomato leaves. Spore suspension (10^5^ spores/mL) was drop inoculated on upper surface of sterilized leaves of tomato under *in vitro* conditions; (**B)** Lesion diameter of WT and corresponding mutants observed on tomato leaves. Observations were made four days post inoculation; (**C)** Disease severity on chickpea plants to screen impaired virulence in *B. cinerea* mutants (T-DNA tagged mutant, complemented mutant and gene deletion mutant) in comparison to WT. **(D)** Conidia germination rate of respective strains on onion epidermis after incubation at 22 °C was evaluated after 24 hpi, (**E)** Penetration ability of strains was assessed at 48 hpi. Data are presented as mean ± SD. Bars showing different letters indicate significant differences according to the Duncan’s multiple comparison tests (P < 0.05). (**F)** Tissue colonization of onion epidermis observed after 48 h of inoculation. Spore suspension (10^5^ spores/mL) was drop inoculated on hydrophobic surface of sterilized tissue. Epidermis was stained with cotton blue in lactophenol for microscopic examination. Scale bars represent 20 µm. Arrows indicate penetration of hyphae in epidermis. Experiment was performed in five replicates and was repeated three times.

Detached tomato leaves were used to assess the virulence of complemented mutant and deletion mutant in comparison to T-DNA tagged or insertional mutant and WT. Disease symptoms in form of necrotic lesion were observed in WT and *bcKLP-7* ^COM^. In case of BCM-29 and ∆*bcKLP-7* necrotic zones were not clear and were restricted to a smaller surface area (Fig. 7A). Non-significant difference in mean lesion diameter was observed in WT and *bcKLP-7* ^COM^; and between BCM-29 and ∆*bcKLP-7*. In case of WT and *bcKLP-7* ^COM^, significantly two times larger lesion diameters were observed as compared to BCM-29 (P < 0.05) (Fig. 7B). Virulence assay was also carried out on chickpea plants. The disease severity by BCM-29 and ∆*bcKLP-7* significantly reduced in comparison to WT and *bcKLP-7* ^COM^. It was found that *bcKLP-7* ^COM^ restored its functional ability to cause disease. The disease severity in *bcKLP-7* ^COM^ was comparable with that of WT (86%) (P < 0.05) (Fig. 7C). The disease severity observed was five folds higher in case of WT than BCM-29 and ∆*bcKLP-7*. Due to gene deletion in ∆*bcKLP-7*, disease severity was observed to be 15.8% (Fig. 7C).

### Mutant BCM-29 and *∆bcKLP-7* are impaired in plant colonization

It was observed that due to *KLP-7* disruption, BCM-29 showed significantly reduced and delayed conidia germination rate (43.2%) as compared to WT (98%) (P < 0.05) (Fig. 7D). We found that ∆*bcKLP-7* was largely impaired in its conidia germination and tissue colonization abilities. At 48 hpi, notable differences were observed in conidia germination rate among the strains. While 98% conidia germinated in WT, only 42.2% conidia germination was observed in ∆*bcKLP-7* and this difference was found to be significant (P < 0.05). The percentage of conidia germination in *bcKLP-7* ^COM^ was similar to that of WT (Fig. 7D). WT and *bcKLP-7* ^COM^ produced secondary infectious hyphae and penetrated the epidermis at 48 hpi. Further, in case of *bcKLP-7* ^COM^, hyphae were able to penetrate the host tissues and dense tissue colonization was observed similar to that of WT. Also, *bcKLP-7* ^COM^ regained its lost abilities to germinate and colonize the epidermal surface in a similar manner as that of WT. The penetration rate in BCM-29 and ∆*bcKLP-7* was considerably reduced (40% of WT, Fig. 7E) (P < 0.05). At 48 hpi, BCM-29 showed very few hyphae formation and WT produced secondary infectious hyphae in order to colonize the onion surface. Although, the hyphal growth of BCM-29 was irregular and remained short, they were still able to grow on onion epidermis after 48 hpi (Fig. 7F).

### Virulence factors determination

**Figure 8:**
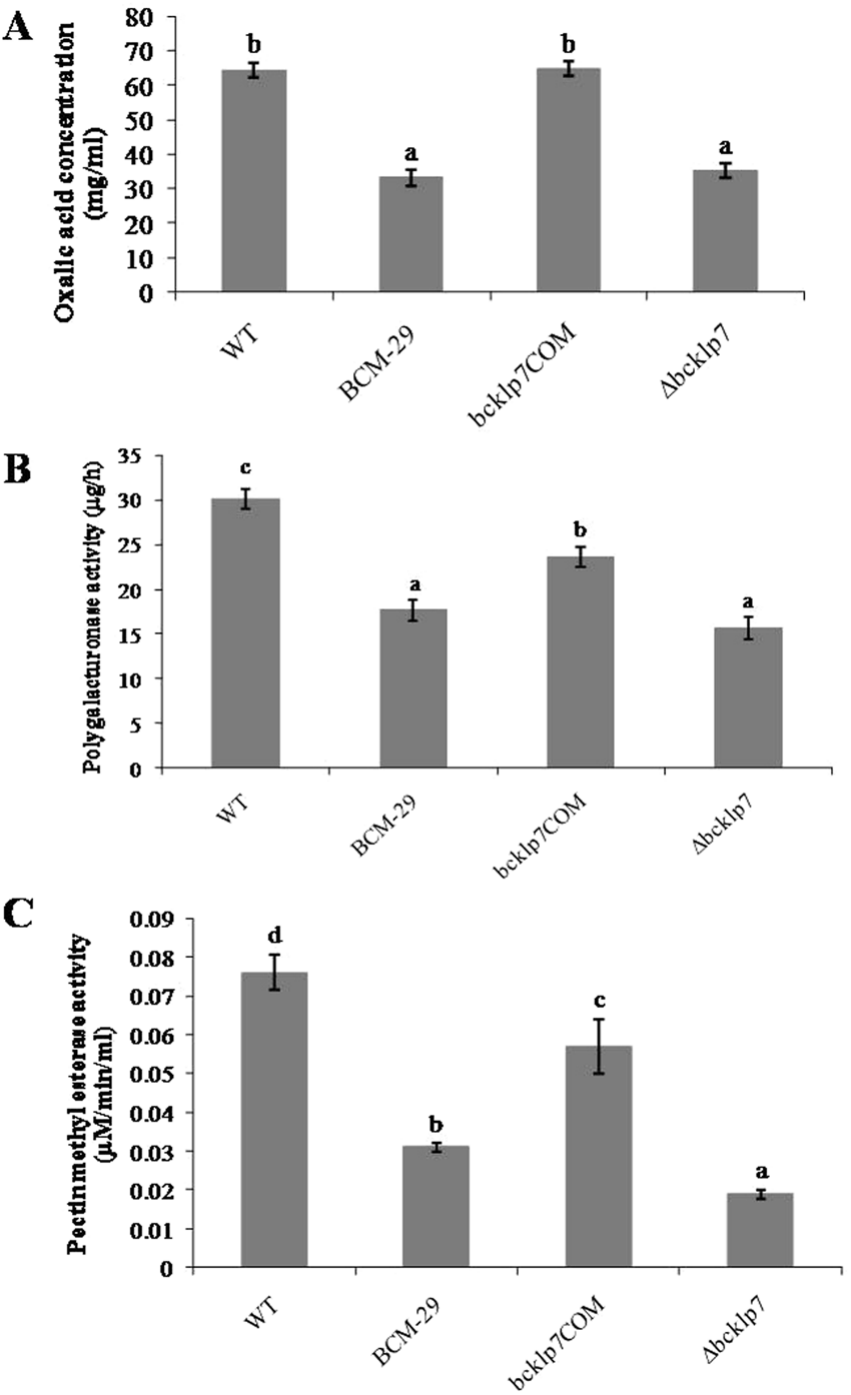
Virulence factors determination. (**A)** Oxalic acid concentration in culture filtrates of WT, BCM-29, *bcKLP-7* ^COM^ and *∆bcKLP-7* incubated for 10 days; (**B)** and (**C)**. The activities of cell wall degrading enzymes PG and PME in WT and corresponding mutants incubated for ten days. Data is presented as mean ± SD. Bars showing different letters indicate significant differences according to the Duncan’s multiple comparison tests (P < 0.05).

A significant two fold decrease in OA accumulation was found in case of BCM-29 as compared to WT (P < 0.05). *bcKLP-7* ^COM^ secreted significantly higher OA (64.8 mg/ml) in comparison to BCM-29. Non-significant differences were observed between WT and *bcKLP-7* ^COM^ (Fig. 8A). The activity of PG and PME was found to be significantly higher in case of WT and *bcKLP-7* ^COM^ as compared to mutants, BCM-29 and ∆*bcKLP-7* (Fig. 8B,C) (P < 0.05). Although, the differences observed in the activity of PG were statistically similar in case of BCM-29 and ∆*bcKLP-7*, non-significant differences were observed for PME activity **(**Fig. 8B,C).

## Discussion

ATMT has been extensively used in different fungi to carry out genetic manipulations^25, 27–30^. Transformation with T-DNA that does not exhibit homology with the fungal genome results in heterologous integration DNA. This enables the use of transforming DNA as an insertional mutagen to disrupt genes, and eventually assist in the study of host-pathogen interactions^31^.

In this study, ATMT was used to generate virulence mutants of *B. cinerea* to identify novel factors required during pathogenesis. This approach helped in generating 800 monokaryotic transformants, out of which 200 transformants exhibited a range of pathogenicity defects. All the mutants displayed stable phenotype and were able to grow on selective medium containing 100 μg/ml of hygromycin even after five successive generations of growth on non-selective medium. The mitotic stability was consistent with T-DNA integration into the chromosomal DNA^32–36^. T-DNA insertion was also analysed by using *hph* gene specific primers. After confirming T-DNA integration events, virulence assay was conducted on chickpea plants. One of the mutants (BCM-29) that displayed quantitative defects in disease development and exhibited consistent low virulence on several rounds of pathogenicity screening was chosen in order to identify the potential factors responsible for its impaired virulence.

Identification of tagged gene was largely fulfilled by TAIL-PCR strategy. The location sequence at which the T-DNA integrated was identified by walking through the known flanking insert end of borders. BLAST analysis of the sequence showed similarity with BC1G_00851.1 with homology to *KLP-7* gene that encodes a kinesin protein. Based on *in silico* analysis carried out by Schoch *et al*.^26^ a total of 11 kinesin gene families have been identified and it was suggested that *KLP-7* clade constitutes a unique fungal subgroup of “truncated” UNC-104-like proteins that may constitute a new “subfamily”. *KLP-7* motor domain is highly conserved in the UNC-104 kinesin family. We observed that *KLP-7* homologs are found only in the subphylum Pezizomycotina of Ascomycota. *KLP-7* shared sequence similarities with kinesin protein from other fungal genus.

Kinesins and myosins are generally involved in transport of organelles in fungal hyphae^37, 38^. Kinesin motors have also been identified to play a vital role in hyphal growth of the plant pathogen, *U*. *maydis* ^39^. Out of ten kinesins analysed by them, conventional kinesin (Kinesin-1) and Unc104/Kif1A-like kinesin (Kinesin-3) have been found to be essential for hyphal growth. Similarly, our study also revealed that disruption of *KLP-7* gene leads to impaired hyphal growth in *B. cinerea*, which might have resulted in the decreased virulence of BCM-29 on chickpea plants. In order to evaluate the role of *KLP-7* in virulence, complementation and targeted deletion was carried out. Complementation included the ectopic integration of *KLP-7* gene in BCM-29 whereas complete coding DNA sequence of *KLP-7* was removed in the WT strain to generate deletion mutants. Relative transcript abundance was evaluated among WT and other mutants by qRT-PCR.

High relative expression of *KLP-7* was observed in complemented mutant *bcKLP-7* ^COM^ as compared to BCM-29 and ∆*bcKLP-7*. The expression level was found significantly similar in complemented mutant *bcKLP-7* ^COM^ and WT. Virulence assay conducted on chickpea plants showed that per cent disease severity in the complemented mutant *bcKLP-7* ^COM^ was significantly higher (five folds) in comparison to the corresponding insertional mutant BCM-29. Impaired virulence in BCM-29 was rescued upon complementation. Disease progression pattern in *bcKLP-7* ^COM^ was similar to WT while targeted deletion resulted in reduced virulence as observed in case of BCM-29. *In vitro* virulence assay carried out on tomato leaves also followed similar trend. Lesion formation was maximum in WT isolate followed by *bcKLP-7* ^COM^ and no lesions were formed by BCM-29 and ∆*bcKLP-7* until four days of incubation under high humidity conditions. Also the rate of conidia formation was lower in BCM-29 and ∆*bcKLP-7* with respect to WT which was restored in *bcKLP-7* ^COM^.

Tissue colonization ability of WT and mutants was assessed on onion epidermis. The hyphae of WT and *bcKLP-7* ^COM^ ramified in order to colonize the underlying host tissue. Similar intensity of colonization was not observed in case of BCM-29 and ∆*bcKLP-7*. The data confirmed that complementation of the gene restored the functional properties that were lost due to insertional mutagenesis.

*KLP-7* gene has been known to play role in hyphal growth^39^. Our morphological analysis showed that colony growth rate was slower in BCM-29 and ∆*bcKLP-7* with respect to WT and *bcKLP-7* ^COM^. In this study, scanning electron micrographs depicted irregular and constricted hyphae in BCM-29 and ∆*bcKLP-7* as compared to uniform (smooth) and broader hyphae in WT and *bcKLP-7* ^COM^. In addition to this, conidia size and conidiation were also affected by *KLP-7* disruption. On reinstating the gene in insertional mutant, the conidia size and rate of conidia formation were found equivalent to WT. Hyphal deformation accompanied by slower growth and altered colony phenotype has also been reported in other fungal species deficient in kinesin expression^40–42^. Therefore, it is likely that *KLP-7* is involved in regulation of hyphal growth, conidia size and conidiation in *B. cinerea*.

In this study, the insertional mutant exhibited significant reduced virulence on host plant tissue. Prominent lesion formation on tomato leaves was induced by WT isolate followed by *bcKLP-7* ^COM^ while BCM-29 and ∆*bcKLP-7* produced significantly smaller necrotic spots. Virulence assay conducted on chickpea plants also showed a similar trend. As it was observed that BCM-29 and ∆*bcKLP-7* were impaired in conidia production, the number of conidia was increased to compensate the defect. However, no alterations in virulence level were noted. Impaired virulence in BCM-29 was rescued upon complementation. Therefore, we infer from this study that *KLP-7* might have a role in virulence of *B. cinerea*.

In order to trace the reduced pathogenic behaviour, development of fungal hyphae inside the host tissue was determined by conducting infection assay on onion epidermis. It was observed that conidia germination rate was conspicuously lower in BCM-29 and ∆*bcKLP-7*. This reduced germination rate was also accompanied with low penetration events. While, WT and *bcKLP-7* ^COM^ produced long ramifying hyphae to invade the tissue, hyphal growth was stunted in BCM-29 and ∆*bcKLP-7*. Penetration events are known to be controlled by turgor pressure generated in hyphae. Hence, there is a possibility that lower turgor pressure in smaller hyphae of disruption mutants have contributed to lower their penetration ability. Similar observations of reduced virulence have been recorded for *kin 2*, a conventional kinesin in *Ustilago maydis* ^43^, wherein deletion mutants produced short hyphae and were unable to move the cytoplasm to the tip compartment. The phenotype of this mutant was attributed to a secretion defect and its inability to enlarge basal vacuoles. *Kin 2* mutants showed notably reduced mating as well as virulence that illustrated the crucial role of kinesin during the infection process^37, 41^. Impaired virulence observed on *KLP-7* disruption in this study can thus be explained by reduced hyphal growth which consequently hampers the ability of fungus to colonize the host surface.

A pathogenic fungus secretes armour of virulence factors to invade the host tissue^44^. Extracellular secretion of these factors facilitates infection. Although several factors have been studied, only a few are explicitly involved in virulence. OA is a potential virulent factor in necrotrophs that results in acidic environmental conditions which favour fungal invasion^3^. EndoPG and PME have also been shown to play a role in the pathogenesis of *B. cinerea* ^22, 44^. Therefore analysis of OA, PG and PME as potent virulence factors were analyzed in the present study to check whether deletion of *KLP-7* also affected the release of these factors. We found OA, PG and PME activity were higher in case of WT and *bcKLP-7* ^COM^ as compared to mutants, BCM-29 and ∆*bcKLP-7*. In both the cases the activities of the three factors were severely decreased. We found that complementation of the full length gene could reinstate this function hence indicating role of *KLP-7* in regulation of their secretion. This suggests that *KLP-7* is an important factor in their secretion thereby influencing the virulence of *B. cinerea*. Although, no such role of kinesins has ever been contemplated, we hypothesize that due to impaired hyphal growth and inability to move cytoplasm to the tip compartment the secretion of these factors into host environment may be affected. However, stating the exact role of kinesins in secretion of these virulence factors warrant thorough investigation.

Till date, no reports are available to corroborate the role of fungal *KLP-7* in virulence. Using ATMT, gene tagging, complementation strategies and targeted gene deletion, we were able to identify the role of *KLP-7* in virulence. Our results indicate the importance of *KLP-7* in regulating vegetative growth, conidiation, secretion of virulence factors and hence a critical role in virulence of *B. cinerea*. The study will contribute to an enhanced understanding of fungal pathogenesis and assist in the development of alternative control approaches for the management of *Botrytis* grey mould disease.

## Materials and Methods

### Strains and culture conditions

*B. cinerea* parental strain was collected from chickpea field of Govind Ballabh Pant University, Pantnagar, Uttarakhand, India during cool and humid climatic conditions. Infected chickpea leaves were surface sterilized in 1% sodium hypochlorite for three minutes followed by 70% ethanol treatment for five minutes. The leaves were finally washed 3-4 times in autoclaved distilled water under aseptic conditions and were inoculated on potato dextrose agar (PDA, per liter: 200 g potato infusion, 20 g dextrose, 15 g agar). Plates were incubated at 22 °C for seven days. Fungal colonies that were cottony white in appearance were selected and transferred on fresh PDA plates. Microscopic analysis of mycelia structure and conidiophores was carried out at 40X magnification using Nikon Eclipse 80i microscope. The isolate was confirmed on the basis of molecular identification using *B. cinerea* specific primer C729^+^/729^–^ (Table S3)^45^ that amplifies a 700 bp product. The culture was maintained at 22 °C. The field isolated strain was named as wild type (WT) and was used for transformation of *B. cinerea*.

*Agrobacterium tumefaciens* LBA 4404 was procured from Indian Agriculture Research Institute (IARI), Indian Type Culture Collection, New Delhi and maintained on Luria Bertani (LB, per liter: 10 g casein enzymic hydrolysate, 5 g yeast extract and 10 g sodium chloride) medium supplemented with streptomycin (250 µg/mL) at 28 °C.

### Plasmid construction

T-DNA binary vector pDJW5^27^ (Size: 9,851 bp) carrying the hygromycin B phosphotransferase (*hph*) gene cassette under the control of *Aspergillus nidulans* trpC promoter and terminator between the right and left borders (RB/ LB) was used for generating insertional mutants. For complementation of *KLP-7* in BCM-29, pBIF2-*EGFP* vector was used^46^. For this purpose, a 1,491 bp long fragment of *KLP-7* was cloned downstream of gpdA promoter at *Nco*I site using *KLP-7*-*Nco*I-F and *KLP-7*-*Nco*I-R primers. The orientation of the *KLP-7* gene was confirmed using *KLP-7*-*Nco*I-F and *EGFP*-R primers (Table S3). The resulting plasmid was named as pBIF2-*KLP-7*-*EGFP*.

### Minimum inhibitory concentration of hygromycin B

Mycelium of WT was inoculated on PDA medium containing a dilution series of hygromycin B from 10–200 µg/mL. The lowest concentration at which no fungal growth occurred was considered to be lethal for WT and thus was used for selection of transformants. For primary and secondary selection of transformants 50 µg/ml and 100 µg/mL hygromycin B was used respectively.

### ATMT

*Agrobacterium tumefaciens* strain LBA 4404 harbouring the modified binary vector pDJW5 was used to transform conidia of *B. cinerea* ^28, 47^. The bacterial culture was grown in LB medium till optical density reached 0.5-0.6 at 600 nm. Freshly harvested fungal conidia were suspended in induction medium (IM, per liter: 10 g KNO_3_, 1 g K_2_HPO_4_, 0.50 g MgSO_4_, 0.1 g Thiamine, 0.0050 g biotin supplemented with pectin as the only carbon source) and adjusted to 1 × 10^5^ spores/mL using a haemocytometer^2^. Different co-cultivation conditions such as bacterial and fungal spore ratio, concentration of acetosyringone (As), co-cultivation temperature, pH of induction medium and use of filter discs were optimised. Aliquots of each bacterial suspension and spore suspension were mixed and spread over a nylon membrane in an IM-agar plate containing 200 µM of As. After incubation at 22 °C for two days, filters were transferred to PDA medium containing hygromycin B (50 µg/mL) and cefotaxim (200 µM). Transformants obtained after 15 days of incubation at 22 °C were individually transferred to fresh selection medium. For further selection of transformants, conidia were individually collected using a compound microscope and grown on PDA containing hygromycin B (50 µg/ml) and cefotaxim (200 µM). After four days of incubation at 22 °C, the emerging colonies were transferred to PDA medium supplemented with 100 µg/mL hygromycin B and were used for further experiments.

### Pathogenicity assay

For this purpose WT and transformants were tested under Controlled Environmental Conditions (CEC) at National Phytotron Facility, IARI, New Delhi, using whole plant screening technique^48^. Seeds of susceptible chickpea (*Cicer arietinum*) variety P-256 (procured from National Seed Corporation, IARI, New Delhi) were raised in 6″ pots filled with autoclaved mixture of sand, peat and vermiculite in a ratio of 1:2:1. Six seedlings per pot were transferred to CEC maintained at 20 ± 1 °C and ~1500 lux light intensity, to acclimatize for 24 h. Three weeks old plants were inoculated by spraying a conidial suspension (1 × 10^5^ conidia/mL) of *B. cinerea* till runoff. Control plants were treated with sterile distilled water. The inoculated plants were covered with polythene bags to avoid dislodging of spores and to maintain 100% relative humidity. Disease severity was monitored for ten days and per cent disease severity was calculated as follows^49^.

$$ \% \,{\rm{disease}}\,\,{\rm{severity}}=\frac{{\rm{Number}}\,\,{\rm{of}}\,\,{\rm{leaves}}\,\,{\rm{with}}\,\,{\rm{symptoms}}}{{\rm{Total}}\,\,{\rm{number}}\,\,{\rm{of}}\,\,{\rm{leaves}}}\times 100$$

For the detached leaf assay, sterile tomato leaves (Pusa Ruby variety) were harvested, rinsed in water, dried and placed in Petri plates^8^. On each leaf, 10 µL of spore suspension (1 × 10^5^ spores/ml) was inoculated. After four days of incubation at 22 °C, the disease lesion on the leaf surface was evaluated and compared with that of WT. The experiment was performed in ten replicates and was repeated twice.

### Characterization of transformants

WT and selected transformants were grown on PDA and PDA containing hygromycin B (100 µg/mL) respectively at 22 °C for seven days under white light (12 h light/12 h darkness) for conidiation and to study their growth response in terms of colony diameter. For sclerotia production strains were grown on PDA and incubated for 3 weeks at 22 °C in darkness. In order to determine the dry weight of mycelia, all the strains were grown in potato dextrose broth (PDB, per liter: 200 g potato infusion and 10 g dextrose) for seven days at 22 °C. The fungal mycelium was harvested from culture fluid by filtration using Whatman filter paper no. 1. The mycelial pellet hence obtained was repeatedly washed with sterile distilled water and dried at 80 °C for 48 h. The dry weight of the mycelium was calculated by using the following formula:

$${\rm{Dry}}\,{\rm{weight}}=({\rm{weight}}\,{\rm{of}}\,{\rm{filter}}\,{\rm{paper}}+{\rm{mycelium}})-({\rm{weight}}\,{\rm{of}}\,{\rm{filter}}\,{\rm{paper}}).$$

### Oxalic acid and hydrolytic enzyme assay

For OA activity, WT and transformants were grown in 100 mL flasks containing 15 ml of P﻿DB. OA was quantified in culture filtrate. The concentration of OA was calculated by extrapolating absorbance against a standard curve and was expressed in mg/mL^50^. In order to estimate PG and PME activity, WT and transformants were grown in IM supplemented with pectin as the only carbon source. Cultures were incubated for 10 days at 20 °C and the filtrate was used as enzyme extract. PG activity was determined by measuring the amount of reducing sugars (galacturonic acid) released from non-esterified pectin hydrolysed in the reaction mixture^22^. The reaction mixture containing l ml of 0.5% pectin in 0.05 M citrate phosphate buffer at pH 5.0 and 0.5 mL of crude enzyme extract, was incubated at 30 °C for 3 h^51^. One unit of PG was determined by measuring the amount of enzyme under assay conditions that catalyses the release of 10 μg D-galacturonic acid/h. PME activity was determined as described previously^52^. Amount of acid was calculated by titrating the reaction mixture against 0.01 N NaOH. The enzyme activity was expressed as the acid released/min/mL of enzyme preparation.

### Molecular confirmation of transformation events (PCR, Southern hybridization and mitotic stability of transformants)

Presence of T-DNA in the corresponding transformants was confirmed by amplifying *hph* gene using gene specific primers (Table S3). Genomic DNA was extracted from WT and transformants using standard procedures^53^. PCR was carried out using 50 ng/µL of genomic DNA in 50 µL reaction containing 3.0 U of Taq polymerase (Genei, India) and 10 mM each dNTP’s. The cycler conditions employed were as follows: initial denaturation at 94 °C for 5 min followed by 30 cycles: denaturation at 94 °C for 30 s, annealing at 55 °C for 40 s, extension at 72 °C for 2 min followed by a final extension of 5 min at 72 °C. The PCR product was separated on 1.0% agarose gel stained using 1 mg/mL ethidium bromide (Himedia, India) and visualized under UV light (UVP, BioDoc-It Imaging System, USA). Southern hybridisation was performed to determine the frequency and randomness of T-DNA integration. For this purpose, genomic DNA (20 µg) was first digested with *Xho*I, the fragments were separated on 0.8% agarose gel for 8 h and then transferred to a nylon membrane (Millipore, USA). A hygromycin probe was generated by PCR using *hph*-F and *hph*-R primers and radiolabelled with α-^32^P-dCTP (BRIT, India) using random oligonucleotide primer labelling procedure following manual instructions (Promega). The blot was hybridized with the probe for 16 h at 65 °C. Finally, autoradiograms were prepared using Phospho-Imager intensifying screen. To determine the mitotic stability of transformants, selected transformant was cultured on PDA medium in the absence of antibiotic i.e. hygromycin B for five successive generations. Resistance of these mono-conidial cultures to hygromycin B was tested by growing them again on PDA amended with hygromycin B (100 µg/mL).

### Isolation and identification of T-DNA tagged gene via Thermal asymmetric interlaced polymerase chain reaction (TAIL-PCR)

T-DNA insertion flanking sites in the transformants were identified using TAIL-PCR. This technique includes three nested PCR reactions utilizing three border specific primers either left border (LB1, LB2 and LB3) or right border (RB1, RB2 and RB3) along with an arbitrary degenerate primer (AD3)^54, 55^ **(**Table S4 **)**. The reaction conditions and thermal cycling settings were used as described^31^. Primary reactions consisted genomic DNA of transformant as template and either LB1 or RB1 with AD3. For secondary PCR, amplified product from primary PCR and the appropriate nested primers (LB2/RB2 with AD3) were used. For tertiary PCR, amplified products from secondary PCR and nested primers LB3/RB3 with AD3 were used. Amplified products from primary, secondary and tertiary PCR reactions were analysed on 1.2% agarose gel electrophoresis. A single PCR product was generally obtained following the third round of PCR. DNA fragment from the tertiary PCR reaction showed a decrease in length (between the second and third PCRs) and was consistent with primer positions on the T-DNA. Tertiary PCR product which was assumed to correspond to T-DNA junction was purified using Qiagen Gel Extraction kit (Qiagen), cloned in pGEM-T easy vector and sequenced. The sequence obtained was then analysed using BLAST and *Botryotinia fuckeliana* database.

### Cloning, selection of transformants and complementation

CDS of *KLP-7* tagged in BCM-29 (Acc No. AY230421.1) was retrieved from *B. fuckeliana* database and amplified using *Pfu* polymerase (Fermentas) from the cDNA of WT using gene specific primers (Table S3). Amplified product was cloned into pGEM-T easy vector and sequenced. Full length sequence of *KLP-7* gene was sub-cloned in pBIF2-EGFP vector at NcoI site. This construct was named pBIF2-*KLP-7*-EGFP.

For complementation, pBIF2-*KLP-7*-EGFP construct was transformed in insertional mutant BCM-29 using ATMT^28, 47^ as mentioned in previous section. Transformants were selected on PDA medium containing geneticin (1 mg/mL) and cefotaxim (200 µM). Further, to screen transformants an additional selection marker i.e. enhanced green fluorescent protein (*egfp*) was used and transformants were selected using fluorescence microscopy and PCR using eGFP primers. Ectopic integration of pBIF2-*KLP-7*-EGFP in *bcKLP-7* ^*COM*^ was checked by PCR using *KLP-7*-NcoI-F and *KLP-7*-NcoI-R primers (Table S3 **)**.

### Western blot analysis

To determine the expression of GFP fused with KLP-7 protein, WT *B. cinerea*, BCM-29 and *bcKLP-7* ^*COM*^ were grown in appropriate selective media (as mentioned in previous section) for 7 days at 22 ± 2 °C temperature. For protein isolation fungal mycelia were frozen in liquid nitrogen, homogenised and extracted with lysis buffer [(50 mM Tris-HCL (pH-7.5), 100 mM NaCl, 1% Triton X-100, 1 mM DTT, 10% glycerol)]. Protease inhibitor cocktail (Calbiochem, Millipore, Germany**)** and phosphatase inhibitor cocktail (Biobasic, Canada) was added to the mixture, vortexed and centrifuged. Protein content was separated by SDS-polyacrylamide gel. ﻿1﻿2 µg of protein was loaded on a 10% SDS-PAGE and proteins were transferred to PVDF membrane (Mini Trans-Blot® Electrophoretic Transfer Cell (Bio-Rad). Blot was probed with 1:20000 dilutions of polyclonal anti-GFP antibody (Abcam) for 16 hours at 4 °C. After 3 washings, blot was probed with secondary Goat Anti-Rabbit IgG antibody (1:10000 dilutions) conjugated with horseradish peroxidase (HRP). Blot was developed with Clarity^TM^ Western ECL substrate kit (BioRad) using Hyper processor^TM^ (Amersham, Biosciences).

### Targeted *KLP-7* gene deletion

For this purpose pBIF vector was used^46^. A 1038 bp long 5′flanking and 1030 bp long 3′ flanking region of *KLP-7* gene was amplified with a primer pair, *KLP-7*KO5′*Eco*RI-F, *KLP-7*KO5′*Eco*RI-R and *KLP-7*KO3′*Xho*I-F, *KLP-7*KO3′*Xho*I-R respectively. Both 5′ and 3′ flanking regions of target sequences were cloned at *Eco*RI and *Xho*I on respective ends of *hph* cassette. The directionality of 5′ and 3′flanking region fused with *hph* cassette (1,503 bp) was confirmed by using *KLP-7*KO5′*Eco*RI-F, *hph*-R and *hph*-F, *KLP-7*KO3′ *Xho*I-R primers respectively. The gene disrupted cassette fused with *hph* was named as pBIF-*KLP-7*KO (3,571 bp) and was transformed by ATMT in WT. Mutants were selected on PDA supplemented with hygromycin. Deletion of *KLP-7* gene was also confirmed by PCR using primer pairs *KLP7*-NcoI-F and *KLP7*-NcoI-R (Table S3).

### Functional role of putative *KLP-7*

In order to confirm the functional role of *KLP-7* gene in virulence: WT, BCM-29, *bcKLP-7* ^COM^ and ∆*bcKLP-7* were screened *via* whole plant virulence assay on chickpea under CEC as described previously. Cytological studies were performed to monitor the infection related morphogenesis of fungal hyphae as described^8^. The sterilized onion epidermal surfaces were first excised in small pieces (1 ﻿cm^2^) and then inoculated with equal conidial suspension (1 × 10^5^ conidia/mL). Control was inoculated using sterile distilled water. The inoculated tissue was placed in sterilized and moistened Petri plates at 22 °C. At 48 hour post inoculation (hpi) the tissue was stained by applying a drop of 0.01% lactophenol cotton blue. The surface of the cleared inoculated tissue was examined for germination and penetration of conidial germ tubes. The sections were photographed under a compound light microscope (Nikon Eclipse 80i). For fluorescence microscopy, fresh mycelia were harvested. The microscopic slides were prepared by mounting the mycelia in glycerol. The fluorescence patterns were inspected under FITC filters fixed in fluorescence microscope (Carl Zeiss, AxioScope A.1).

### Scanning electron microscopy (SEM)

To observe morphology of spores and to determine differences in hyphal structure- WT, BCM-29, *bcKLP-7* ^COM^ and ∆*bcKLP-7* were grown on PDA for four days at 20 °C. Fresh mycelia (1 mm^2^) were harvested in triplicates and were fixed in modified Karnovsky’s fixative for 4 h at 4 °C. Thereafter, samples were washed in 0.1 M sodium phosphate buffer (pH 7.2) three to four times and dehydrated through graded acetone series (30 min each) at 4 °C. The material was dried at critical point and coated with gold (100A° thickness) to eliminate charging and mounted on aluminium stubs. The sections were observed under JSM-84 SEM microscope.

### Quantitative Real Time PCR (q-RT-PCR)

To study the expression of putative *KLP-7* in WT, BCM-29, *bcKLP-7* ^COM^, ∆*bcKLP-7*, RNA was extracted from ten days old mycelia of *B. cinerea* samples (500 mg) using TRIZOL reagent according to the manufacturer’s instructions (Invitrogen-USA). Quality and quantity of RNA was analysed by using Agilent 2100 Bioanalyzer (Agilent Technologies Inc., Palo Alto, CA). The extracted RNA was normalized and cDNA was synthesized. Further cDNA was subjected to real-time quantitative PCR using gene specific primer and SYBR Green I with ROX reference dye in an ABI 7500 Real-Time PCR System (Applied Biosystems). The forward and reverse primer pairs for the *KLP-7* and BcAct (Actin) gene were used to check the expression (Table S3). The reaction mixture was heated at 95 °C for 3 min and then subjected to 40 PCR cycles of 95 °C for 15 s, 60 °C annealing for 30 s and 72 °C extension for 30 s and the resulting fluorescence was monitored. The heat dissociation curves confirmed that a single PCR product was amplified for each gene. The melting temperatures were 81.3 °C and 85.3 °C for the PCR products of the *KLP-7* and BcAct gene, respectively. The level of target mRNA, relative to the mean of reference housekeeping gene (BcAct), was calculated by the comparative Ct method.

### Bioinformatics, homology and phylogenetic analysis of Kinesin protein

All sequence information used in this study were obtained from NCBI. To identify *KLP-7* homologs, GeneBank (http://www.ncbi.nlm.nih.gov/BLAST) database were searched using the BLAST algorithm. *KLP-7* homologs were intensively analyzed by NCBI database fungal genomes (including three Oomycetes) using the BLAST matrix tool, which plots the BLAST results by taxonomic distribution^56^. Sequence alignment was done using MULTALIN with BLOSUM 62 matrix with gap penalty of 10 for insertion and 5 for extension and generation of bootstrapped phylogenetic trees were performed with MEGA7^57, 58^. A total of 54 homologs from diverse organism groups such as fungi, plants, insects, animals and bacteria were used for homology and to construct phylogenetic tree by using BLASTp tool and MEGA 7 software, respectively.

### Statistical analyses

The data were analyzed using the Statistical Package for Social Sciences version 16 (SPSS Inc., Wacker Drive, Chicago, IL) for windows. All results are given as mean ± standard deviation. Differences between the individual mean were compared using Duncan’s multiple comparison tests (P < 0.05).

## Electronic supplementary material

Supplementary information
