# Supplementary material for: A Botrytis cinerea KLP-7 Kinesin acts as a Virulence Determinant during Plant Infection
Source: Sci Rep. 2017 Sep 6;7:10664. doi: 10.1038/s41598-017-09409-5 (PMC5587557; doi:10.1038/s41598-017-09409-5)
Supplement: Supplementary file 1 — Supplementary information [file 41598_2017_9409_MOESM1_ESM.pdf]

A *Botrytis cinerea KLP-7* Kinesin acts as a Virulence Determinant during Plant Infection

Pamil Tayal[1], Sumit Raj[2], Esha Sharma[1], Manoj Kumar[2], Vikram Dayaman[2], Meenakshi Dua[3], Nidhi Verma[2], Abhimanyu Jogawat[2], Rupam Kapoor[1‡] and Atul Kumar Johri[2§]

[1]Department of Botany, University of Delhi, Delhi-110 007, India,

[2]School of Life Sciences, [3]School of Environmental Sciences, Jawaharlal Nehru University, New Delhi-110 067, India.

[*]Running title: Role of *Kinesin in virulence*

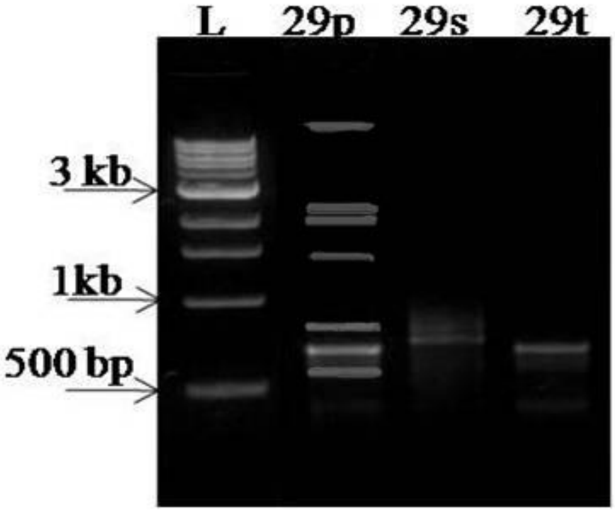

Figure S1: **Agarose gel electrophoresis of thermal asymmetric interlaced polymerase chain reaction (TAIL-PCR)** to show primary (p), secondary (s) and tertiary (t) PCR products of BCM-29 transformant amplified using AD3, RB1, RB2 and RB3 primers respectively.

Figure S2

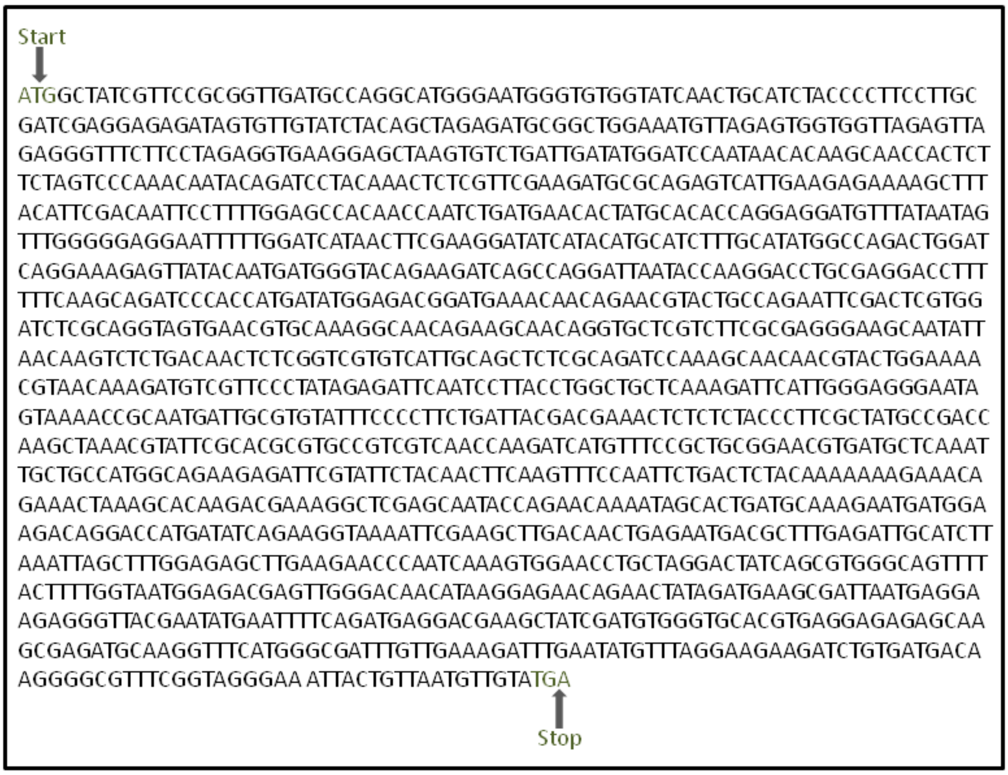

Figure S2: The complete CDS of *KLP-7*. Gene is 1491 bp long.

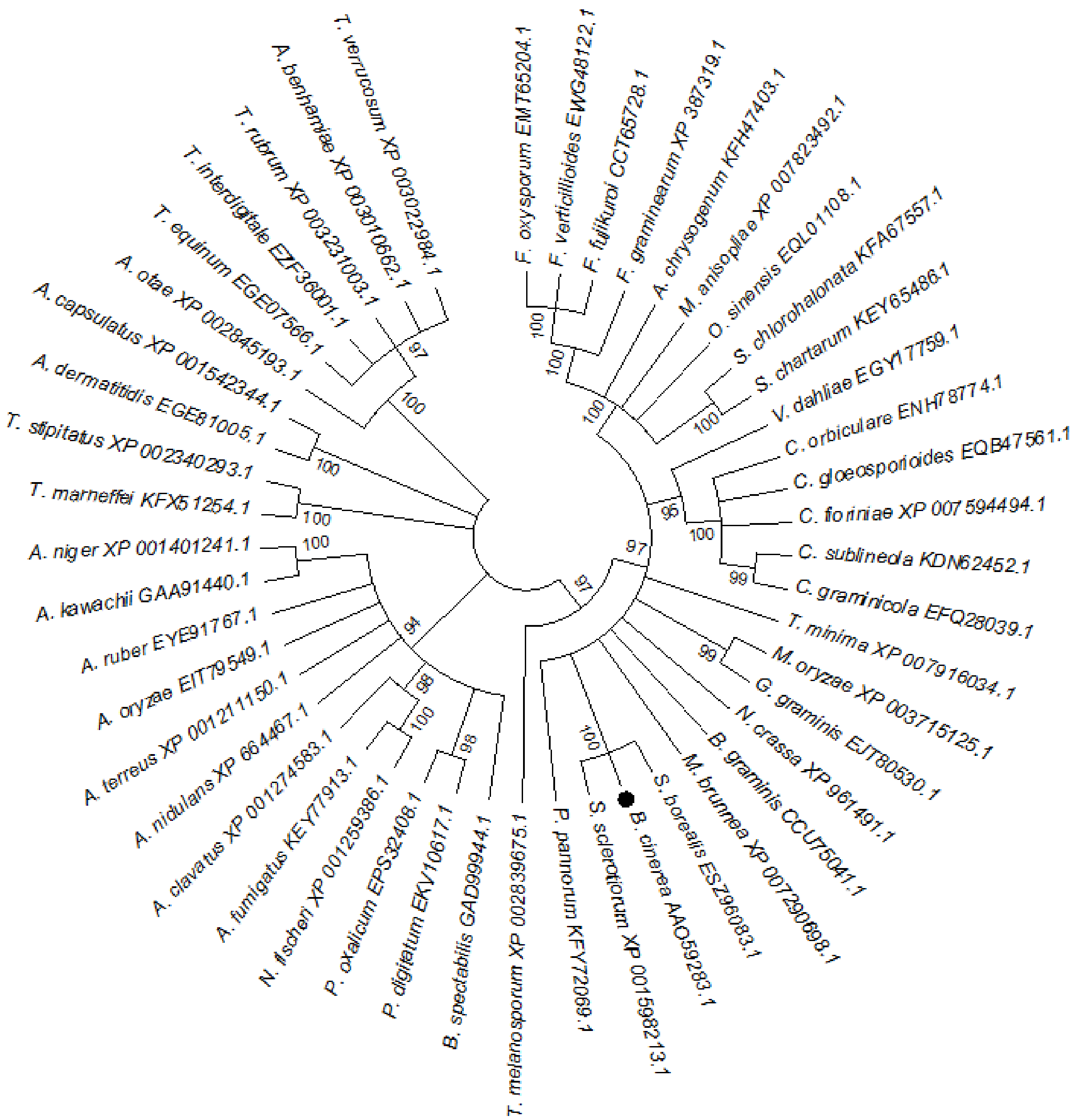

Figure S3. Phylogenetic relationship of *KLP-7* with other fungal kinesins containing motor domain from Pezizomycotina.

KLP-7 Protein names are indicated in Table S1. The evolutionary history was inferred using the Neighbor-Joining method. The *tree* is drawn to scale, with branch lengths in the same units as those of the evolutionary distances used to infer the phylogenetic tree. The evolutionary distances were computed using the Maximum Composite Likelihood method and are in the units of the number of base substitutions per site. All positions containing gaps and missing data were eliminated from the dataset (Complete Deletion option). Phylogenetic analyses were done by using MEGA 7.

Table S1: Conserved *KLP-7* Homologs in Subphylum Pezizomycotina

Phylum	Subphylum	Species	Locus Name	Protein Length	E-value	Query Coverage/ Sequence Identity in %
**Oomycota**		*Phytophthora infestans*	None			
		Phytophthora ramorum	None			
		Phytophthora sojae	None			
**Ascomycota**	**Pezizomycotina**	*Botrytis cinerea*	AAO59283	496	0	100/100
		Sclerotinia sclerotiorum	XP_001598213	596	0	98/89
		Sclerotinia borealis	ESZ96083	597	0	99/ 87
		Trichophyton equinum	EGE07566	538	4e-171	95/57
		Marssonina brunnea	XP_007290698	637	3e-144	97/71
		Togninia minima	XP_007916034	531	7e-138	84/70
		Colletotrichum orbiculare	ENH78774	587	2e-132	97/69
		Pseudogymnoascus pannorum	KFY72069	599	4e-132	86/68
		Colletotrichum sublineola	KDN62452	583	2e-130	94/70
		Colletotrichum graminicola	EFQ28039	590	3e-130	94/67
		Pestalotiopsis fici	XP_007834412	651	1e-129	94/67
		Colletotrichum higginsianum	CCF36340	583	2e-128	94/68
		Verticillium dahlia	EGY17759	605	1e-127	94/68
		Pseudogymnoascus destructans	ELR05622	603	2e-126	97/66
		Talaromyces stipitatus	XP_002340293	661	9e-124	94/66
		Byssochlamys spectabilis	GAD99944	649	7e-123	92/64
		Aspergillus clavatus	XP_001274583	655	7e-122	93/63
		Fusarium verticillioides	EWG48122	615	1e-121	95/66
		Aspergillus terreus	XP_001211150	627	3e-121	94/65
		Neosartorya fischeri	XP_001259386	655	4e-121	94/65
		Magnaporthe oryzae	XP_003715125	614	7e-117	93/63
		Aspergillus niger	XP_001401241	658	6e-116	94/63
		Neurospora crassa OR74A	XP_961491	676	2e-113	94/61
		Bipolaris maydis	AAO59294	598	3e-105	95/55
	**Saccharomycotina**	*Candida albicans*	None			
		Candida glabrata	None			
		Candida guilliermondii	None			
		Candida lusitaniae	None			
		Candida tropicalis	None			
		Eremothecium gossypii	None			
		Kluyveromyces lactis	None			
		Kluyveromyces waltii	None			
		Saccharomyces castellii	None			
		Saccharomyces cerevisiae 288C 2007	None			
		Saccharomyces cerevisiae RM11	None			
		Saccharomyces cerevisiae YJM789	None			
		Lodderomyces elongisporus	None			
		Pichia stipitis	None			
		Saccharomyces bayanus	None			
		Saccharomyces kluyveri	None			
		Saccharomyces kudriavzevii	None			
		Saccharomyces mikatae	None			
		Saccharomyces paradoxus	None			
		Yarrowia lipolytica	None			
	**Taphrinomycotina**	*Pneumocystis carinii*	None			
		Schizosaccharomyces japonicas	None			
		Schizosaccharomyces pombe	None			
**Basidiomycota**		*Coprinus cinereus 1*	None			
		Cryptococcus neoformans serotype B	None			
		Cryptococcus neoformans serotype D	None			
		Laccaria bicolour	None			

	Phanerochaete chrysosporium	None			
	Ustilago maydis	None			
**Chitridiomycota**	*Batrachochytrium dendrobatidis*	None			
**Microsporidia**	*Antonospora locustae*	None			
	Encephalitozoon cuniculi	None			
**Pucciniomycotina**	*Sporobolomyces roseus*	None			
**Pucciniomycotina**	*Phycomyces blakesleeanus*	None			
	Rhizopus oryzae	None			

Table S2: Summary of amino acids identity (%), query cover (%) and E-values between *B. Cineria kinesin* and other fungal, plant, insects, animals and bacteria kinesin like proteins.

Phylum	Species	GenBank Accession Number	Protein Length	E-value	Query Coverage/ Sequence Identity in %
**Fungi**	*Botrytis cinerea*	AAO59283	496	0	100/100
	Sclerotinia sclerotiorum	XP_001598213	596	0	98/89
	Sclerotinia borealis	ESZ96083	597	0	99/ 87
	Trichophyton equinum	EGE07566	538	4e-171	95/57
	Marssonina brunnea	XP_007290698	637	3e-144	97/71
	Togninia minima	XP_007916034	531	7e-138	84/70
	Colletotrichum orbiculare	ENH78774	587	2e-132	97/69
	Pseudogymnoascus pannorum	KFY72069	599	4e-132	86/68
	Colletotrichum sublineola	KDN62452	583	2e-130	94/70
	Colletotrichum graminicola	EFQ28039	590	3e-130	94/67
	Pestalotiopsis fici	XP_007834412	651	1e-129	94/67
	Colletotrichum higginsianum	CCF36340	583	2e-128	94/68
	Verticillium dahlia	EGY17759	605	1e-127	94/68
	Pseudogymnoascus destructans	ELR05622	603	2e-126	97/66
	Talaromyces stipitatus	XP_002340293	661	9e-124	94/66
	Byssochlamys spectabilis	GAD99944	649	7e-123	92/64
	Aspergillus clavatus	XP_001274583	655	7e-122	93/63
	Fusarium verticillioides	EWG48122	615	1e-121	95/66
	Aspergillus terreus	XP_001211150	627	3e-121	94/65
	Neosartorya fischeri	XP_001259386	655	4e-121	94/65
	Magnaporthe oryzae	XP_003715125	614	7e-117	93/63
	Aspergillus niger	XP_001401241	658	6e-116	94/63
	Neurospora crassa OR74A	XP_961491	676	2e-113	94/61
	Bipolaris maydis	AAO59294	598	3e-105	95/55
	Candida albicans	XP_716594.1	972	2e-39	30/53
	Candida albicans	XP_716334.1	904	4e-27	47/38
**Plant**	*Klebsormidium flaccidum*	GAQ88188.1	1547	3e-44	30/44
	Glycine soja	KHN27080.1	991	2e-42	35/36
	Aegilops tauschii	EMT25397.1	926	2e-37	33/34
	Triticum urartu	EMS63470.1	853	6e-38	24/39
	Noccaea caerulescens	JAU28489.1	300	2e-41	59/44
	Spinacia oleracea	KNA10715.1	1306	1e-40	24/44
	Glycine max	XP_014625430.1	1063	3e-42	33/36
	Trifolium subterraneum	GAU23670.1	331	2e-43	72/40
	Corchorus olitorius	OMP01569.1	2191	2e-42	28/40
	Arabidopsis thaliana	AAC24096.1	1032	4e-38	35/35
	Arabidopsis thaliana	BAB11329.1	1032	5e-40	25/49
	Arabidopsis thaliana	OAO94080.1	1035	5e-40	25/49
	Arabidopsis thaliana	NP_172389.2	1010	7e-38	35/35
	Oryza sativa	XP_015623546.1	1265	3e-40	35/43
	Oryza sativa	CAE05519.1	1094	7e-39	24/37
	Oryza sativa	AAO72688.1	614	2e-33	69/33
**Insect**	*Lasius niger*	KMQ92801.1	653	1e-68	37/49
	Bactrocera cucurbitae	XP_011184284.1	1721	7e-61	17/45
	Clastoptera arizonana	JAS05530.1	389	2e-53	62/62
	Drosophila yakuba	XP_002092166.1	1670	2e-51	20/52
	Drosophila persimilis	XP_002026608.1	1275	1e-51	23/63
	Drosophila melanogaster	NP_001303353.1	1661	2e-51	20/52
	Ceratitis capitata	XP_012160298.1	1666	2e-51	18/62
	Bactrocera latifrons	JAI33985.1	583	5e-53	56/63
	Stomoxys calcitrans	XP_013109773.1	1816	1e-50	14/62
	Musca domestica	XP_005180931.2	1665	1e-50	15/62
	Corethrella appendiculata	JAB59061.1	1683	1e-52	18/56
	Amyelois transitella	XP_013200952.1	1654	4e-51	22/50
	Rhagoletis zephyria	XP_017483990.1	1688	4e-51	18/62
Animal	*Oesophagostomum dentatum*	KHJ97730.1	505	3e-54	54/50
	Oncorhynchus kisutch	XP_020333734.1	352	7e-53	58/48
	Fulmarus glacialis	KFW10749.1	640	2e-52	46/61
	Necator americanus	XP_013297951.1	1579	7e-52	21/50
	Ancylostoma ceylanicum	EPB79993.1	1537	1e-51	20/50
Bacteria	*Escherichia coli*	WP_083580879.1	457	6e-06	27/22

Table S3: Primers used for the identification of transformants, cloning, complementation, targeted deletion and expression of *KLP-7*.

**Name**	**Sequences (5'-3')**	**Purpose**
Hph-F	GAAGATCTTACCATGAGCCTGAACTCACCG	To confirm the presence of T-DNA by amplifying *hph* gene
Hph-R	CGAGCTCCTATTCGCCCCTGGACGA	
KLP7-Nco1-R	CACACCATGGCTACAACATTAACAGTAATTTCCCTACCG	Cloning, complementation and confirmation of deletion *KLP-7* gene
KLP7-Nco1-F	CACACCATGGGTTTACGAGAGACCCTTCATGTACC	
eGFP-F	ACGGCGACGTAAACGGCCAC	To select complemented BCM-29cc
eGFP-R	GTGATCGCGCTTCTCGTTGG	
KLP7KO5'EcoRI-F	GGAATTCCAATCCAAATGCCATCGAGCA	Cloning 5'flanking region of KLP7 gene
KLP7KO5'EcoRI-R	GGAATTCCAACCGCAATGATTGCGTGTA	
KLP7KO3'Xho1-F	CCGCTCGAGCGGGGTAACAGTTTTCAAATGGGAGG	Cloning 3'flanking region of *KLP-7* gene
KLP7KO3'Xho1-R	CCGCTCGAGCGGAAGCTATCTCAGTTCTCCTGGCC	
HPHF	TTCCGGAAGTGCTTGACATT	directional cloning
HPHR	TTCTACACAGCCATCGGTCC	
RT *Kin* F	TCTCGCAGGTAGTGAAC	For expression of *KLP-7* using q-RT-PCR
RT *Kin* R	TTGAGCAGCCAGGTAAGG 3'	
BcActF (Actin)	TCTGTCTTGGGTCTTGAGAG	For expression of actin using q-RT-PCR
BcActR (Actin)	GGTGCAAGAGCAGTGATTTC	
C729^{+}	AGCTCGAGAGAGATCTCTGA	To confirm *B.cinerea* isolate
C729^{-}	CTGCAATGTTCTGCGTGGAA	

Table S4 : Primers used for TAIL-PCR.

Primer	Sequence (5’ - 3’)
LB1	GGGTTCCTATAGGGTTTCGCTCATG
LB2	CATGTGTTGAGCATATAAGAAACCCT
LB3	GAATTAATTCGGCGTTAATTCAGT
RB1	GGCACTGGCCGTCGTTTTACAAC
RB2	AACGTCGTGACTGGGAAAACCCT
RB3	CCCTTCCCAACAGTTGCGCA
AD3	NTCGASTWTSGWGTT

W = A or T; N = A, T, C or G; S = G or C
